# Genetic distance as an alternative to physical distance for definition of gene units in association studies

**DOI:** 10.1186/1471-2164-15-408

**Published:** 2014-05-28

**Authors:** Cristina Rodriguez-Fontenla, Manuel Calaza, Antonio Gonzalez

**Affiliations:** Laboratorio de Investigacion 10 and Rheumatology Unit, Instituto de Investigacion Sanitaria - Hospital Clinico Universitario de Santiago, Santiago de Compostela, Spain

## Abstract

**Background:**

Some association studies, as the implemented in VEGAS, ALIGATOR, i-GSEA4GWAS, GSA-SNP and other software tools, use genes as the unit of analysis. These genes include the coding sequence plus flanking sequences. Polymorphisms in the flanking sequences are of interest because they involve cis-regulatory elements or they inform on untyped genetic variants trough linkage disequilibrium. Gene extensions have customarily been defined as ± 50 Kb. This approach is not fully satisfactory because genetic relationships between neighbouring sequences are a function of genetic distances, which are only poorly replaced by physical distances.

**Results:**

Standardized recombination rates (SRR) from the deCODE recombination map were used as units of genetic distances. We searched for a SRR producing flanking sequences near the ± 50 Kb offset that has been common in previous studies. A SRR ≥ 2 was selected because it led to gene extensions with median length = 45.3 Kb and the simplicity of an integer value. As expected, boundaries of the genes defined with the ± 50 Kb and with the SRR ≥2 rules were rarely concordant. The impact of these differences was illustrated with the interpretation of top association signals from two large studies including many hits and their detailed analysis based in different criteria. The definition based in genetic distance was more concordant with the results of these studies than the based in physical distance. In the analysis of 18 top disease associated loci form the first study, the SRR ≥2 genes led to a fully concordant interpretation in 17 loci; the ± 50 Kb genes only in 6. Interpretation of the 43 putative functional genes of the second study based in the SRR ≥2 definition only missed 4 of the genes, whereas the based in the ± 50 Kb definition missed 10 genes.

**Conclusions:**

A gene definition based on genetic distance led to results more concordant with expert detailed analyses than the commonly used based in physical distance. The genome coordinates for each gene are provided to maintain a simple use of the new definitions.

**Electronic supplementary material:**

The online version of this article (doi:10.1186/1471-2164-15-408) contains supplementary material, which is available to authorized users.

## Background

Genes are the unit of analysis or interpretation of multiple genetic association studies. However, multiple operational definitions of genes coexist in current use. Some are restricted to the coding sequence but, most often, they are extended to include flanking sequences because they contain polymorphisms that are informative of variation in the coding sequence trough linkage disequilibrium (LD) or polymorphisms that are themselves functional by involving regulatory sequences. Here, we have addressed the definition of these gene extensions for application in gene- or pathway-based association studies, gene-based interaction analysis and interpretation of large numbers of top association signals for meta-analysis or for gene- and pathway- enrichment analysis.

Gene- or pathway- based association studies [1–8] consider the genes, not the individual SNPs, as the units of analysis. Association statistics for the genes are obtained by combining the statistics corresponding to the SNPs mapping to each of them. In this way, it becomes possible to identify genes with multiple independent SNPs contributing to the trait but lacking significant association on their own. The same considerations apply to pathway- or gene-set analyses, where the association signals from the genes in a pathway are combined. A similar situation appears in interaction analyses where the objective is to identify pairs of genes contributing to a trait in a way that deviates from the simple addition of their independent effects [9, 10]. This type of analysis can be done at the individual SNP level but this is very sensitive to small variations in the study, and analysis at the gene or pathway level has been advocated as more reproducible [9–11]. In addition, extended gene definitions can be useful in analysis that by considering many top association signals find it impractical a detailed analysis of each of them. For example, when it is necessary to decide if associations from a large number of studies are coincident or not in the same gene [12], or when interpreting multiple association signals [13, 14].

In all these situations, genes have been operationally defined as the coding sequence plus a fixed physical distance in each direction. Length of the extensions has been from 0 to 500 [5, 7] Kb, but most often of 20 [8, 13, 14] or 50 [1–4, 9] Kb. This is a practical solution that is used because of its simplicity, but this definition is subjective and not fit for many genes. Here, we propose a definition of genes that is equally easy to apply and has the advantage of including genetic distance in place of physical distance. Genetic distance is the relevant one because it determines LD between polymorphisms [15–18] and, therefore, the information that SNPs in the extensions provide about un-typed variation in the coding or regulatory sequences. Genetic and physical distances are not interchangeable because the correspondence between the two is very variable along the genome [15–18]. We took genetic distances as standardized recombination ratios (SRR) from the deCODE recombination map [16], which is the most accurate available. The new extended gene definitions were compared with definitions based on physical distances to illustrate their advantages. They are made available in a text file with genome coordinates to facilitate their use.

## Results

### Setting a SRR threshold

**Figure 1:**
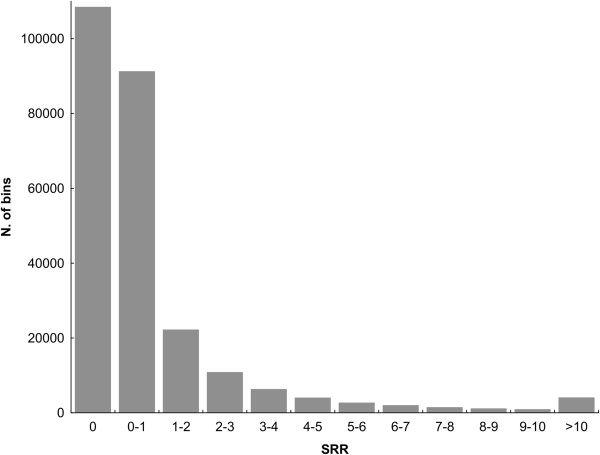
**Distribution of the standardized recombination rate (SRR) in the human genome.** Number of 10 Kb bins from the deCODE recombination map [16] within each interval of SRR values.

It is well known that the recombination rate is very irregular along the human genome [15–18]. This irregularity leads to a skewed distribution of SRR along the genome (Figure 1) [16] including a large fraction of bins, 42.6%, with no recombination (SRR = 0) and 78.4% of the bins with less than the average (SRR < 1). Therefore, most of the recombination takes place in the remaining 21.6% bins. Analysis of the SRR distribution showed that extensions of genes based on an SRR ≥ 2 have a median physical length of 45.3 Kb (IQR = 22.9-90.2 Kb). This median length is similar to the most common physical distance extension used until now, which is of 50 Kb. The SRR ≥ 2 is only found in a minor fraction of bins, 12.9%. The remaining 87.1% of the 10 Kb bins showed lower SRR. No detailed optimization of the SRR was attempted preferring to keep the simplicity of an integer value.

### Comparison of genetic and physical distance based gene definitions

**Figure 2:**
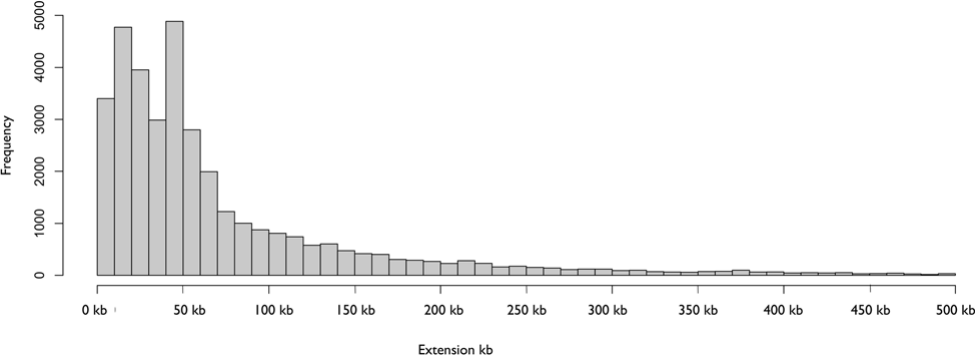
**Length distribution of the 36 044 gene extensions according to the SRR ≥ 2 rule.** The 5′ and 3′ extensions for each gene have been separately considered. All followed the SRR ≥ 2 rule except for 2669 of genes near telomeres and centromeres, where information is incomplete and that were replaced by the median length of extensions in their chromosomes; most of them in the 40–50 Kb range.

Concordance between the median length of the extensions based on SRR ≥ 2 and the ± 50 Kb rule made possible a direct comparison. However, the new definitions obtained here account for recombination and are variable (Figure 2), not uniform. They go from less than 10 Kb (8.8% of the extensions) to more than 500 Kb (1.2% of the extensions). The distribution of extension lengths implies that most gene boundaries are discordant between the two definitions. In fact, only 21.3% of the extensions obtained with one definition are within ± 10 Kb of the obtained with the other, and even less frequently (6.1%) when the two extensions of a gene are considered simultaneously.

**Figure 3:**
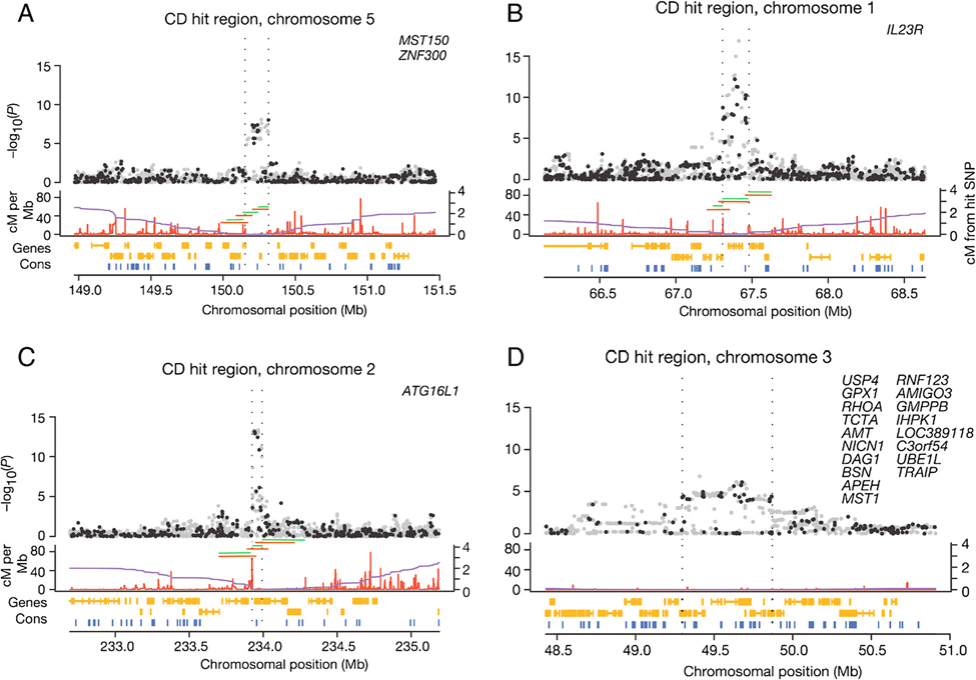
**Relevant genes in loci from the WTCCC GWAS depending on gene definitions.** Image modified form Figure five of the WTCCC 2007 GWAS paper with permision [19]. Horizontal lines corresponding to the genes overlaping with the region of association have been added in the middle panel of **A) B)** and **C)**, in red for the ± 50 Kb rule and in green for the SRR ≥ 2 definition. No lines were added for panel **D)**. Association region in each locus is limited by vertical dotted lines. The upper panel represents the SNPs (black dots, genotyped; grey dots, imputed) in function of position (X axis) and association (Y axis = -log_10_(*P*)). Middle panel, centimorgans per Mb estimated from Phase II HapMap. The purple line shows the cumulative genetic distance (in cM) from the hit SNP. Lower panel, known genes in orange, top track shows plus-strand genes and the middle track shows minus-strand genes in condensed format. Below these tracks, sequence conservation in 17 vertebrates. Information in middle and lower panels is from the UCSC Genome Browser. Positions are in NCBI build-35 coordinates. Known genes in the hit region according the WTCCC paper are listed in the upper right part.

We have used two large GWAS with multiple associated loci to illustrate differences between the two gene definitions. However, these analyses should not be confused with an attempt to replace detailed analysis of GWAS results. First, we used the interpretation of 18 top association signals from the 2007 WTCCC GWAS [19]. The authors of this study gave lists of relevant genes for each associated locus based on analysis of the associated SNPs and LD around the top signal. These lists include from 0 to 23 genes. The SRR ≥ 2 definition led to lists that were more concordant with the WTCCC GWAS than the obtained with the ± 50 Kb definition (Table 1 and Additional file 1: Table S1 [20]). All the genes selected by the WTCCC authors were also included when applying the two definitions, but in some loci the gene definitions led to consider some extra genes. Specifically, the SRR ≥ 2 definition included additional genes in one locus, whereas the ± 50 Kb definition included additional genes in 12 of the 18 loci (P = 0.00015 for the comparison of fully concordant loci). In more detail, six loci included an extra gene according to the ± 50 Kb rule (an example shown in Figure 3A); four loci included two extra genes with the ± 50 Kb definition (two of these loci shown in Figure 3B and C); an additional locus included 3 extra genes in the list obtained with the ± 50 Kb definition (Table 1). The remaining locus was the unique in which the three lists were discordant. This locus is particularly difficult because it shows a very low recombination rate and, therefore, a very wide region of association with ill-defined limits (Figure 3D). In addition, it shows a high density of genes implying large differences when applying alternative criteria. Overall, there were 107 genes in the 18 association regions according with detailed analysis done by the WTCCC authors. The definition based on genetic distances led to fully concordant results except for the difficult locus, where no criterion can be considered certain (Figure 3D). In contrast, the definition based on ± 50 Kb included 26 additional genes (P = 0.00025 for the comparison of the number of extra genes). Nine of these extra genes were from the difficult locus in chromosome 3, but there were 17 extra genes in other loci. This example illustrates the very good concordance between *post-hoc* detailed analysis of each locus done by the WTCCC authors and the simple overlap with gene definitions based on genetic distances. It also illustrates the differences between this definition and the based on a fixed physical distance.

**Table 1:** **Number of genes in association regions of the WTCCC GWAS top hits**[19]

Chromosome	Disease^b^	WTCCC	SRR ≥ 2^c^	± 50 Kb^c^
*5p13*	CD	0	-	-
*10q24*	CD	1	-	-
*10q25*	T2D	1	-	-
*9p21*	CAD	2	-	-
*10q21*	CD	3	-	-
*16q12*	CD	4	-	-
*16q12*	T2D	1	-	+ 1
*5q33*	CD	2	-	+ 1
*1p13* ^a^	RA	7	-	+ 1
*1p13* ^a^	T1D	7	-	+ 1
*16p13*	T1D	8	-	+ 1
*16p12*	BD	9	-	+ 1
*1p31*	CD	1	-	+ 2
*2q37*	CD	1	-	+ 2
*18p11*	CD	1	-	+ 2
*12q24*	T1D	15	-	+ 2
*12q13*	T1D	26	-	+ 3
*3p21*	CD	18	+ 7	+ 9
	Total	107	+ 7	+ 26

^a^These two loci overlap.

^b^ *CD* = Crohn’s disease, *T2D* = Type 2 diabetes, *CAD* = Coronary artery disease, *RA* = Rheumatoid arthrtitis, *T1D* = Type 1 diabetes, *BD* = Bipolar disorder.

^c^SRR ≥ 2 for the gene definition extended to reach a cumulative SRR ≥ 2 in each direction; and ± 50 Kb for gene definition extended to this length in each direction. Only changes in the number of genes, not in their identity, were observed between the three lists:- no differences with the genes highlighted by the WTCCC authors; + number of additional genes beyond the highlighted by the WTCCC authors. A full list of genes in each loci is available as Additional file 1: Table S1.

The second study used to illustrate differences between the gene definitions is a large GWAS that included a selection of putative functional candidate genes for many of the associated loci [21]. The authors of this study used two criteria to identify these genes. The two were based in SNPs that are in high LD (r^2^ > 0.8) with the top associated SNP and with predictable functional relevance because they disrupt the protein sequence, nsSNPs, or the expression of a nearby gene, *cis*-eQTLs. The search extended to the more than 3 000 genes mapping 1 Mb around the 75 top associated signals. It led to 43 functional candidates (Table 2). These putative functional candidates were prioritized relative to other genes in the loci and the aim of our current test has been to evaluate the capacity of the two gene definitions to highlight them. We found that the SRR ≥ 2 definition performed better than the definition based on ± 50 Kb (Table 2). The difference was due to a larger number of genes failing to be highlighted by the latter approach. In more detail: the two methods missed the same candidate genes in 3 loci, the SRR ≥ 2 definition missed an additional candidate, but the ± 50 Kb missed other 7 candidate genes (P = 0.028; Table 2). In this way, the SRR ≥ 2 definition missed 9.3% of the putative functional candidates, whereas the ± 50 Kb definition missed 23.3% of them.

**Table 2:** **Functional candidate genes that are missed depending on the gene definition**

Chromosome^a^	Phenotype^b^	van der Harst et al.	SRR ≥ 2^c^	± 50 Kb^c^
nsSNP				
*1q23*	MCHC	*OR6Y1, OR10Z1, SPTA1*	-	*OR6Y1*
*1q44*	RBC	*TRIM58*	-	-
*6p21*	MCH	*HFE*	*HFE*	*HFE*
*6p21*	RBC	*HLA-DQA1*	-	-
*10q11*	MCV	*MARCH8*	-	-
*11q13*	MCV	*RPS6KB2*	-	-
*11q13*	HB	*ARHGEF17*	-	-
*12q24*	HB	*SH2B3*	-	-
*12q24*	MCV	*ACADS*	-	-
*16q22*	RBC	*CTRL, PSMB10*	-	*CTRL, PSMB10*
*19p13*	MCV	*UBXD1, NUDT19*	*UBXD1*	*UBXD1*
*22q11*	MCV	*YDJC*	*YDJC*	-
*22q12*	MCH	*FBXO7, TMPRSS6*	-	-
	∑ nsSNP	18	-3	-5
eQTL				
*4q27*	MCV	*CCNA2*	-	-
*6p23*	MCH	*GMPR*	-	-
*6p21*	RBC	*HLA-DQA1/HLA-DQA2*	*HLA-DQA2*	*HLA-DQA2*
*8p11*	MCHC	*C8orf40*	-	-
*10q11*	MCV	*MARCH8*	-	-
*11p15*	HB	*AKIP1/C11orf16, NRIP3*	-	*NRIP3*
*11q13*	MCV	*RPS6KB2, PTPRCAP/COROB1*	-	-
*11q13*	HB	*ARHGEF17*	-	-
*15q22*	MCV	*PTPLAD1*	-	*PTPLAD1*
*15q25*	MCHC	*DNAJA4*	-	-
*16q22*	RBC	*DUS2L*	-	*DUS2L*
*17q11*	MCH	*ERAL1, TRAF4*	-	*ERAL1*
*17q12*	RBC	*CDK12*	-	-
*17q25*	HB	*PGS1*	-	-
*18q21*	MCH	*C18orf25*	-	-
*19p13*	MCH	*CALR, FARSA*	-	-
*22q11*	MCV	*UBE2L3*	-	-
*22q13*	MCV	*ECGF1*	-	-
	∑ eQTL	25	-1	-5
	Total	43	-4	-10

^a^Loci in chromosome *17q21* were excluded from analysis because it contains a common inversion polymorphism of approximately 900 kb in populations with European ancestry that shows exceptional LD and inheritance [26].

^b^Phenotypes were: *MHCH* = Mean cell haemoglobin concentration, *RBC* = Red blood cell count, *MCH* = Mean cell haemoglobin, *MCV* = Mean cell volume and *HB* = Haemoglobin.

^c^Genes that did not overlap with the SRR ≥ 2 or the ± 50 Kb definition are indicated: - no differences with the functional candidate genes highlighted by van der Harst *et al*. [20]; genes that were highlighted by van der Harst *et al*. [20] but whose definition did not overlap with the top associated SNP.

Functional candidates were selected in van der Harst *et al*. [20] because they contained nsSNP (upper rows) or were regulated by eQTL (lower rows) in LD with the top associated SNP.

## Discussion

The gene definitions based on genetic distances lead to extensions with different physical lengths, meaning that most gene definitions are discordant from any other based on a fixed length as we have shown for the SRR ≥ 2 and ± 50 Kb. The advantages of the new definition stem from the fact that physical distance is an inaccurate substitute of genetic distance as a measure of the relationships between polymorphisms in the population [15–18]. This has been illustrated by showing a better performance in the interpretation of top association signals of the simple overlap rule based on SRR ≥ 2 definitions than in the traditional ± 50 Kb In consequence, the new gene definitions will improve gene- and pathway-based analysis by definition. The benefits are obtained by shortening gene extensions where recombination is high and by lengthening them where recombination is low.

These gene definitions are not intended for interpretation of top association results in individual GWAS. In every case that a more detailed analysis is worth the extra effort, it should be done. Our choice of two GWAS as examples for illustration of the differences between the two gene definition approaches was motivated by the quality and reproducibility of GWAS, not to predicate the use of gene definitions in this field. The two GWAS were selected because they were of high quality, have found a large number of loci, have done detailed analysis of all the loci and have provided a full description of the genes selected for each of them. These are uncommon characteristics and we were fortunate that the two studies used different approaches for selecting the putative functional genes allowing a more thorough comparison of the ± 50 Kb and the SRR ≥ 2 gene definitions.

Other gene extensions based on genetic distances are possible. We chose the threshold of SRR ≥ 2 because it produced extensions of similar median physical length as the most used in previous studies. It will be inappropriate and arbitrary to compare other SRR thresholds with the ± 50 Kb gene definition because these definitions will have different coverage of the genome and such comparisons will mix two components: differences in coverage and lack of correspondence between genetic and physical distances. By using the SRR ≥ 2 rule we assured an equivalent coverage of the genome and the comparison was focused in the lack of correspondence between the two distances. Later we found that it led to concordant results with detailed *post-hoc* analysis in 17 of 18 WTCCC GWAS associated loci and to inclusion of 90% of 43 functional candidates for red blood cell associated loci. Therefore, this definition seems convenient although we do not claim that more appropriate SRR thresholds could not be found for specific applications at around this value.

Our approach of using genetic distances in place of physical ones is widely applicable; but the gene definitions we provide are only directly applicable to Europeans. Other maps and specific genetic parameters will be necessary to study other ethnic groups. A genetic map for individuals of African ancestry has already been reported [22]. In addition, we have taken genetic distances from the deCODE recombination map [16], but genetic maps based on the decrease of LD can be taken as alternatives. Currently the best of these maps has been produced with HapMap samples [17, 18]. Although the recombination map and the LD based maps have a high degree of correlation, there are differences between them and some gene definitions will be discordant. Both maps were obtained on the NCBI36 genome assembly that has been replaced by more recent ones. However, conversion of the maps to current assemblies will decrease their accuracy and we consider that is more accurate to convert SNP data to the NCBI36 assembly (with liftover in the UCSC browser at http://genome.ucsc.edu/cgi-bin/hgLiftOver, Remap in the NCBI site at http://www.ncbi.nlm.nih.gov/genome/tools/remap, or a similar tool), perform definition of gene units with the SRR ≥ 2 rule and run the intended analyses with the gene- or pathway-units.

We used the RefSeq catalogue of protein coding genes for our analysis [23]. At least other four human gene sets are widely available, all of them different in some aspect although sharing sources of information and methodologies and being more or less interconnected [24, 25]. These sets are in continuous revision to incorporate findings of new experiments and technologies and none claims to be complete or definitive. The RefSeq set has been manually curated after incorporating information from multiple sources. It is considered conservative and trusted and other annotation projects use it as one of their inputs. Among those using RefSeq input, the GENCODE set combines manual and automatic annotation and is more comprehensive by including the transcripts detected in the ENCODE project. However, the number of the RefSeq, UCSC and GENCODE protein coding genes is very similar. Differences between these sets are remarkable only in the number of transcripts per gene and in the number of exons for each gene [24]. For example, the number of transcript per gene is much larger in GENCODE than in RefSeq , with the UCSC set in between. These differences could slightly modify the boundaries of the gene units defined taking the RefSeq set as reference. Therefore, we consider that the provided gene definitions are generally valid and will perform well but would be not fully consistent with other gene sets.

## Conclusions

A definition of genes based on the coding sequence plus extensions whose length is given by genetic distances was shown to lead to more accurate results in the two sets of top association signals analysed. Use of this definition is made as simple as the commonly used until now by the list of gene coordinates on the physical map that is provided.

## Methods

### Baseline gene definitions

The RefSeq collection (UCSC RefSeq hg18) of 18 022 human protein-coding genes in autosome chromosomes and their map positions from the NCBI36 genome assembly, which corresponds to the used by the deCODE recombination map [16], were taken as the bases to which extensions were attached.

### Recombination information

Relative frequency of recombination for each 10 Kb bin of the human genome was obtained as standardized recombination rates (SRR) from the sex-averaged deCODE recombination map [16]. SRR are the result of dividing the recombination rate corresponding to each bin by the overall recombination mean for the genome.

### Gene definitions

A SRR given gene extensions of median length approaching the most commonly used 50 Kb boundary was searched. SRR inside the coding sequence were not considered. Two gene definitions were compared: one based on physical distance and the other based on genetic distance. The first included RefSeq sequences + 50 Kb in each direction; the second, the RefSeq definition extended in 10 Kb bins until the cumulative SRR that gave a similar median length. In this way, two extensions were generated for each RefSeq gene per definition, one for each end, 5′ and 3′. Genes placed near the telomeres and the centromere of each chromosome were incompletely covered in the deCODE recombination map. For the 2 669 genes in this situation, length of extensions based on genetic distances was made equal to the median length of all other extensions for this specific chromosome. These manipulations were done with PERL and Unix scripts that combined data from RefSeq and the deCODE recombination map, established the extension limits and generated the tabulated plain text file including one row per gene, and the columns: chromosome, “left” boundary, “right” boundary and gene name, which is available for download as supplementary material in Additional file 2[20].

### Assessment of the gene definitions

Differences in length of the extensions between the ± 50 Kb and the SRR based definitions were calculated for all genes. In addition, the two gene definitions were applied to two large GWAS identifying multiple loci and using different criteria to highlight associated genes. Firstly, we examined 18 loci associated in the WTCCC GWAS [19]. This study examined about 14 000 patients, 2 000 of each of 7 different major diseases, and 3 000 healthy controls. Findings included 18 independent associations with p < 5×10^-7^ analysed with detail in Figure five of the Nature paper. The authors defined regions of association (indicated by dotted vertical lines) extending until p values of the SNPs returned to background levels and, where possible, to recombination hotspots. We took these 18 loci and generated lists of genes overlapping with the association regions considering that any part of the genes according to the ± 50 Kb or the SRR definitions falling in between the dotted vertical lines was sufficient to count it. Secondly, we considered the 43 functional candidate genes in the 75 loci identified in the GWAS from van der Harst *et al.* analyzing 6 red blood cell quantitative phenotypes in 135 000 subjects [21]. These functional candidates were selected with two criteria: presence of nsSNP or of eQTL with r^2^ > 0.8 with the top associated SNP. One of the loci was excluded from our analyses due to its exceptionally high LD and unique inheritance pattern due to a common inversion polymorphism under positive selection in Europeans of about 900 Kb in chromosome 17q21 [26]. We took the remaining 43 functional candidate genes and tested if they would be included among the highlighted genes using the ± 50 Kb or the SRR gene definitions around the top associated SNP. The study of van der Harst *et al.* included other criteria for prioritizing candidate genes based in analysis of the bibliography or in the physical proximity that we did not consider here. Comparisons of the number of genes failing with each of the two gene definitions were done with the one-tailed Fisher exact test applied to the 2×2 contingency tables.

## Electronic supplementary material

Additional file 1: Table S1: List of genes included in each of the top associated loci from the WTCCC GWAS (Ref 19) by the authors of the study, with the SRR ≥ 2 definition and with the ± 50 Kb rule. This file is available in the Dryad Digital Repository, doi:10.5061/dryad.p58hb, http://doi.org/10.5061/dryad.p58hb. (XLSX 20 KB)

Additional file 2: **Coordinates with the boundaries for the SRR ≥ 2 gene definitions.** Tabulated plain text file including one row per gene, and the columns: chromosome, “left” boundary, “right” boundary and gene name. This file is available in the Dryad Digital Repository, doi:10.5061/dryad.p58hb, http://doi.org/10.5061/dryad.p58hb. (CSV 554 KB)
